# Trends and disparities in cardiometabolic disease prevalence by physical activity among US adults: NHANES 2007–2018

**DOI:** 10.1016/j.ahr.2026.100290

**Published:** 2026-08-03

**Authors:** Zhijing Xu, Jinyu Hu, Jackie Finik, Xiang Qi, Ruotong Liu, Yaguang Zheng, Bei Wu

**Affiliations:** a School of Nursing, Montclair State University, Montclair, NJ 07043, United States; b Department of Family Science and Human Development, College for Community Health, Montclair State University, Montclair, NJ 07043, United States; c New York University Rory Meyers College of Nursing, New York, NY 10065, United States; d New York University Shanghai, Shanghai 200126, China

**Keywords:** Cardiometabolic disease, Physical activity, Health disparities

## Abstract

**Background:**

Cardiometabolic diseases (CMDs) significantly contribute to U.S. morbidity. Physical activity (PA) is associated with lower CMD prevalence, yet trends in CMD prevalence by PA level and associated disparities remain underexplored.

**Methods:**

We analyzed 2007–2018 NHANES data from 26,380 adults aged ≥20 years. CMDs included diabetes, cardiovascular disease (CVD), and chronic kidney disease (CKD; eGFR <60 mL/min/1.73 m^2^ or ACR ≥30 mg/g). PA was categorized as no activity, some/irregular activity, or regular activity. Age-standardized prevalence and survey-weighted logistic regression estimated trends (reference: 2007–2008) and adjusted odds ratios (aORs).

**Results:**

Diabetes prevalence increased, with higher odds in 2013–2014 (OR = 1.27; 95% CI: 1.01–1.60), 2015–2016 (OR = 1.41; 1.08–1.84), and 2017–2018 (OR = 1.45; 1.16–1.80) and an absolute increase of 2.49% (0.85 to 4.13). No significant trends were observed for CKD (absolute change: 0.11%; −1.57 to 1.80) or CVD (0.10%; −1.40 to 1.59). CMD prevalence was highest among those with no activity and lowest among regular activity. Regular PA was associated with lower odds of diabetes (aOR = 0.70; 0.62–0.79), CKD (aOR = 0.74; 0.65–0.83), and CVD (aOR = 0.75; 0.64–0.87); some/irregular activity was associated with lower odds of CVD only (aOR = 0.72; 0.62–0.85). Non-Hispanic Blacks had the highest CKD and CVD prevalence across PA levels; diabetes prevalence was highest among Mexican Americans with regular activity.

**Conclusion:**

Diabetes prevalence increased significantly, while CKD and CVD prevalence remained stable. Guideline-adherent PA is associated with lower CMD odds, yet disparities underscore the need for equity-driven, culturally tailored PA promotion.

## 1. Introduction

Cardiometabolic diseases (CMDs), including diabetes, chronic kidney disease (CKD), and cardiovascular disease (CVD), are major causes of morbidity, mortality, and healthcare burden in the U.S. and globally [1]. Physical inactivity is a key modifiable risk factor for CMDs [2,3], yet sedentary lifestyles remain widespread despite public health efforts [4-6]. PA contributes to weight control, glucose metabolism, blood pressure regulation, and lipid improvement, and adherence to guidelines is associated with lower CMD risk [7,8]. However, whether sub-threshold PA provides protection and how CMD prevalence trends differ by activity level remains unclear.

Prior studies have examined PA and CMD outcomes, but few assessed whether CMD prevalence trends over time differ across PA categories using nationally representative data [9,10]. Moreover, disparities exist: non-Hispanic Blacks and Mexican Americans often report lower PA and higher CMD burden than non-Hispanic Whites [10-13], partly due to structural barriers such as neighborhood walkability and healthcare access. Whether guideline-level PA benefits are consistent across racial and ethnic groups remains unclear. To our knowledge, no prior National Health and Nutrition Examination Survey (NHANES) [14] based study has simultaneously examined temporal trends across PA levels, estimated PA-CMD associations with sequential covariate adjustment, and quantified racial/ethnic disparities within a single analytical framework.

To address these gaps and provide a comprehensive assessment of physical activity-related differences in cardiometabolic disease burden, we used NHANES data from 2007 to 2018 (14) to: (1) examine 12-year temporal trends in the age-standardized prevalence trends of diabetes, CKD, and CVD across PA levels; (2) estimate the cross-sectional associations between PA levels and CMD odds while sequentially adjusting for confounders. (3) assess whether these temporal trends and risk associations vary by race and ethnicity, identifying potential disparities. We hypothesize that higher PA is associated with lower CMD prevalence and odds, and that disparities persist, with higher CMD burden among minoritized groups [13,15,16]. Findings may inform more effective, equitable strategies to reduce CMD burden through PA promotion.

## 2. Methods

### 2.1. Data source and study design

This study analyzed de-identified, publicly available data from six consecutive NHANES cycles (2007–2008 to 2017–2018). NHANES employs a complex, multistage probability sampling design to assess the health and nutritional status of the noninstitutionalized U.S. civilian population through standardized interviews, physical examinations, and laboratory tests. The study followed the Strengthening the Reporting of Observational Studies in Epidemiology (STROBE) reporting guideline [16]. Institutional review board approval was waived as the data were publicly available and de-identified. All NHANES protocols were approved by the National Center for Health Statistics (NCHS) Research Ethics Review Board, with written informed consent obtained from participants.

Our analytical sample included adults aged 20 years or older (n = 34,770). We restricted the analysis to adults aged ≥20 years because NHANES physical activity and laboratory measures (including eGFR) are consistently validated and reported for this age range; individuals aged 18–19 years are few in number and have different survey instruments for some health-related questions. Exclusions were applied sequentially: participants with missing data on any CMD outcome first excluded (n = 1,876), followed by those with missing data on physical activity (n = 5), those reporting > 24 h of PA per day (n = 67), and finally those with missing data on any covariate (6,442). After all exclusions (Fig. 1), the final sample comprised 26,380 participants, which represents approximately 187 million noninstitutionalized US adults when NHANES survey weights are applied.

### 2.2. Study variables

Physical activity was calculated as weighted minutes per week. For each domain (work, transportation, and recreational activities), activity volume was calculated by multiplying the number of days by duration in minutes. Vigorous-intensify activities (vigorous work, vigorous recreational activities) were weighted by a factor of 2 to reflect their greater intensity relative to moderate-intensity activities (moderate work, walking/bicycling for transportation, moderate recreational activities). PA levels [17] were then categorized using Global Physical Activity Questionnaire (GPAQ) guidelines: (1) No-activity: no moderate or vigorous activity; (2) Some/irregular-activity: activity below guideline recommendations; (3) Regular-activity: meeting guideline recommendations (≥150 min/week moderate or ≥75 min/week vigorous activity). Cardiometabolic diseases (CMDs) included: (1) Diabetes: self-reported physician diagnosis [18]; (2) Chronic Kidney Disease (CKD): defined as estimated glomerular filtration rate (eGFR) <60 mL/min/1.73 m^2^ or urinary albumin-creatinine ratio ≥30 mg/g. The estimated glomerular filtration rate (eGFR) was calculated using the revised Chronic Kidney Disease Epidemiology Collaboration (CKD-EPI) equation [19].


eGRF=142∗min(Scrk,1)α∗max(Scrk,1)−1.200∗0.9938Age∗{1.012,iffemale1,ifmale}Scr:Serumcreatinineinmg∕dLk:0.7(females)or0.9(males)α:−0.241(females)or−0.302(males)min(Scrk,1):theminiumnmofScrkor1max(Scrk,1):themaximumofScrkor1

(3) Cardiovascular Disease [20] (CVD): self-reported diagnosis of heart failure, coronary disease, angina, myocardial infarction, or stroke. It should be noted that physical activity was assessed via self-report, which is subject to recall bias and social desirability bias and may result in misclassification of PA levels. Similarly, diabetes and CVD were defined by self-reported physician diagnosis, which may underestimate true prevalence due to undiagnosed cases or recall inaccuracy. Covariates included age (continuous), sex (female, male), race/ethnicity (race and Hispanic origin) (MA: Mexican American, OH: Other Hispanic, NHW: Non-Hispanic White, NHB: Non-Hispanic Black, Other: Other Race), education (less than high school, high school graduate, some college or above), marital status (married, otherwise), employment (employed, unemployed), income-to-poverty ratio (defined as low: <1.30, middle: 1.30–3.49, high: ≥3.50), body mass index (BMI: underweight<18.5, normal 18.5–24.9, overweight 25–29.9, obese≥30 kg/m^2^), depressive symptoms (continuous, measured using the Patient Health Questionnaire-9 [PHQ-9]) [21], drinking status (never, former, current), and smoking status (never, former, current). Detailed information on all study variables, including exact NHANES variable names, labels, survey cycles, and corresponding data repository references, is provided in Supplemental Table 1.

### 2.3. Statistical analysis

All analyses accounted for NHANES’s complex survey design by applying 12-year sample weights and adjusting for clustering and stratification, following NCHS analytic guidance [22], with the exception of the comparison of included and excluded participants, which was conducted using unweighted NHANES sample. The 12-year sample weights were constructed by dividing the full-sample 2-year MEC examination weight (WTMEC2YR) from each cycle by 6, corresponding to the number of survey cycles included in the analysis. All statistical tests in this study were two-sided, with significance set at *P* < 0.05. We acknowledge the potential for type I error inflation given the number of statistical tests conducted across three CMD outcomes and survey cycles. However, all outcomes and comparisons were pre-specified based on a priori hypotheses rather than exploratory data mining. Given the well-established biological relationships between physical activity and cardiometabolic diseases, a formal correction for multiple comparisons was not applied. Importantly, subgroup analyses by race/ethnicity were descriptive (age-standardized prevalence) and did not involve formal statistical testing, limiting the multiple testing concern to the primary regression analyses.

Descriptive statistics present weighted means and standard deviations for continuous variables (compared using design-adjusted Wald F-tests) and unweighted counts with weighted percentages for categorical variables (compared using Rao-Scott χ^2^ tests).

The estimated prevalence of CMD outcomes (diabetes, CKD, and CVD) were age-standardized to the 2010 U.S. Census adult population by the direct method, applying three age bands (20–44, 45–64, and ≥65 years) [23] with fixed weights of 0.460, 0.361, and 0.179, respectively (Supplemental Table 2). For each cycle, age-standardized prevalence was computed as follows: (i) survey-weighted crude prevalence was estimated for each CMD outcome within each combination of age band and physical activity category, accounting for the complex survey design; (ii) each age-specific prevalence estimate was multiplied by its corresponding standard population weight; and (iii) the resulting products were summed within each physical activity category to obtain the age-standardized prevalence estimate for that category, expressed as:

$$P_{std,\;j}=\sum_{i=1}^{3}P_{ij}*W_{i}$$

i: Age group (20–44, 45–64, and ≥65 years);

j: Physical activity category (no activity, some/irregular activity, regular activity);

$P_{i}$: Survey-weighted crude prevalence for age group i;

$W_{i}$: Corresponding standard population weight;

$P_{std}$: Age-standardized prevalence.

Temporal trends were evaluated with survey-weighted generalized linear model (GLM) with a logit link (equivalent to survey-weighted logistic regression) [24], modelling disease status (1 = present, 0 = absent) as the outcome. Survey cycle was treated as a categorical variable, with cycle 2007–2008 as the reference, to assess temporal changes across cycles. To complement the categorical approach and evaluate the overall directionality of change, global trend tests were conducted using the same model structure but replacing the categorical cycle variable with a continuous ordinal variable, coded 1 through 6 corresponding to cycles 2007–2008 through 2017–2018. The odds ratio derived from this ordinal variable represents the change in odds per one-cycle increment (approximately two years).

Associations between PA levels and CMDs were examined using survey-weighted multivariable GLM with a logit link (survey-weighted multivariable logistic regression), with the no-activity group as the reference. Four sequential models were constructed: Model 1 (unadjusted); Model 2 (adjusted for age, sex, and race/ethnicity); Model 3 (Model 2 plus education, marital status, employment, and income-to-poverty ratio); Model 4 (Model 3 plus BMI, depression score, alcohol use, and smoking status). To assess whether race/ethnicity modified the association between PA and each CMD outcome, formal interaction terms (PA x race/ethnicity) were further tested in the fully adjusted model 4 for each outcome, and the statistical significance of the interaction was evaluated using the design-based Wald F-test. Multicollinearity in the regression analyses was evaluated using the variance inflation factor (VIF), with values below 5 generally indicating acceptable levels of multicollinearity. Model fit was assessed using design-based Nagelkerke pseudo-R^2^ statistics computed for each sequential model across all three CMD outcomes.

To evaluate racial and ethnic disparities in weighted CMD prevalence (age-standardized) by PA level, stratified analyses were conducted across racial and ethnic subgroups (MA, NHB, NHW, OH, and Other). The same age-standardization procedure was applied consistently across all subgroup analyses.

To assess potential selection bias introduced by complete-case analysis, sample characteristics (age, sex, race/ethnicity, education, marital status, employment, and income-to-poverty ratio) of included and excluded participants were compared using independent samples *t*-test for continuous variable and Pearson’s chi-square tests for categorical variables on unweighted analytical samples.

To ensure the robustness of our findings, four sensitivity analyses were conducted. First, diabetes was redefined using an objective biochemical measure, with diabetes identified by a glycohemoglobin level of ≥ 6.5%. Second, physical activity was operationalized as a binary goal-status measure: Not-at-goal, defined as not meeting physical activity guideline recommendations (< 150 min/week of moderate-intensity activity or < 75 min/week of vigorous-intensity activity), and At-goal, defined as meeting those recommendations (≥ 150 min/week of moderate-intensity activity or ≥ 75 min/week of vigorous-intensity activity). Third and fourth, to address the concern that BMI and depression score may function as mediators in the PA-CMD pathway, two sequential models were estimated: Model 1 excluding BMI alone while retaining all other covariates from the fully adjusted Model 4 (age, sex, race/ethnicity, education, marital status, employment, ratio of income to poverty, alcohol use status, and smoking status), and Model 2 excluding both BMI and depression score simultaneously while retaining the remaining covariates.

All analyses were performed using RStudio version 4.4.2, with NHANES variable documentation retrieved directly from the CDC repository using the *nhanesA* package; complex survey design specification and survey-weighted analyses were implemented using the *survey* and *srvyr* packages; data manipulation was conducted using *dplyr* and *purrr*; and results were visualized using *ggplot2*.

## 3. Results

### 3.1. Sample characteristics

The weighted study sample represented approximately 187 million US adults, with a mean age of 47.4 (SD = 16.9) years. Distinct sociodemographic gradients were observed across PA levels. Participants reporting no-activity were older (mean age, 53.4 years SD=17.1) and had higher proportions of NHB (13%) or MA (9.5%) individuals. The some/irregular-activity group had higher proportions of high-income (40%) participants. Health profiles progressively worsened with lower activity; obesity prevalence was 48% in the no-activity group compared with 35% in the regular-activity group, paralleled by higher depression scores (Table 1).

### 3.2. Trends in CMD prevalence

Among all participants, age-standardized diabetes prevalence showed a generally increasing pattern across NHANES cycles from 2007–2008 to 2017–2018, with a small dip in 2009–2010 (8.3%; 95% CI, 7.3–9.2) and subsequent increases overall from 9.4% (8.3–10.4) to 10.9% (9.7–12.1) (Fig. 2; Supplemental Table 3). Compared with survey cycle 2007–2008, the odds of diabetes were higher in 2013–2014 (OR = 1.27; 95% CI, 1.01–1.60; *P* = 0.041), and 2015–2016 (OR = 1.41; 95% CI, 1.08–1.84; *P* = 0.013), and 2017–2018 (OR = 1.45; 95% CI, 1.16–1.80; *P* = 0.001), while no significant differences were observed in 2009–2010 or 2011–2012. The global trend test confirmed a statistically significant increasing trajectory, with the odds of diabetes rising by approximately 9% per two-year cycle (OR = 1.09; 95% CI, 1.05–1.13; *P* < 0.001). The absolute increase in diabetes prevalence from 2007–2008 to 2017–2018 was 2.5 percentage points (95% CI: 0.9 to 4.1) (Table 2; Supplemental Table 3).

For CKD, age-standardized prevalence showed no clear linear trend across NHANES cycles in all participants, starting at 13.3% (12.2–14.3) in 2007–2008, dipping to 11.4% (10.3–12.5) in 2009–2010, peaking at 14.2% (12.9–15.4) in 2013–2014, and ending at 13.4% (12.0–14.7) in 2017–2018 (Fig. 2; Supplemental Table 3). Compared with 2007–2008, the odds of CKD were not significantly different across cycles, although a marginally elevated odds was noted in 2013–2014 (OR = 1.16; 95% CI, 1.00–1.34; P = 0.055). Although the global trend test suggested a small but statistically significant increase across cycles (OR = 1.04; 95% CI, 1.01–1.06; P = 0.015), this should be interpreted cautiously given the absence of consistent cycle-specific differences and the fluctuating prevalence pattern observed across the study period. The absolute change in CKD prevalence from 2007–2008 to 2017–2018 was 0.11 percentage points (95% CI: −1.6 to 1.8) (Table 2; Supplemental Table 3).

Similar to the CKD result, for CVD, age-standardized prevalence showed no clear linear trend across NHANES cycle in all participants, starting at 8.6% (7.5–9.6) in 2007–2008, fluctuating between 8.0% and 8.4% in intermediate cycle, and ending at 8.7% (7.6–9.7) in 2017–2018 (Fig. 2; Supplemental Table 3). Compared with 2007–2008, the odds of CVD were not significantly different across survey cycles. Similarly, no statistically significant global trend test was observed in CKD (OR = 1.03; 95% CI, 0.98–1.07; *P* = 0.252). The absolute change in CVD prevalence from 2007–2008 to 2017–2018 was 0.1 percentage points (95% CI: −1.4 to 1.6) (Table 2; Supplemental Table 3).

### 3.3. Associations between PA and CMDs

Compared with the no-activity group (reference), both higher PA levels were associated with significantly lower unadjusted odds of all three CMDs (Model 1, Fig. 3). For diabetes, after full adjustment (Model 4), only regular-activity remained significantly associated with lower odds (adjusted odds ratio [aOR], 0.70; 95% CI, 0.62–0.79; *P* < 0.001); the association for some/irregular-activity was no longer significant (aOR, 0.86; 95% CI, 0.71–1.04; *P* = 0.117). For CKD, after full adjustment, only regular-activity remained significantly associated with lower odds (aOR, 0.74; 95% CI, 0.65–0.83; *P* < 0.001). For CVD, both some/irregular-activity (aOR, 0.72; 95% CI, 0.62–0.85; *P* < 0.001) and regular-activity (aOR, 0.75; 95% CI, 0.64–0.87; *P* < 0.001) remained significantly associated with lower odds after full adjustment. Interaction tests revealed statistically significant effect modification by race/ethnicity on the association between PA and all three CMD outcomes (all *P* for interaction < .001) (Fig. 3).

### 3.4. Racial/ethnic disparities

Among adults reporting no-activity, the highest prevalence of diabetes was observed in NHB (17.6%; 95% CI, 15.9–19.3), while the lowest prevalence occurred in adults of Other Race (13.6%; 10.5–16.6). For those with some/irregular-activity, MA had the highest prevalence (17.2%; 13.6–20.9), whereas NHW had the lowest (8.7%; 7.1–10.4). This pattern persisted among regularly active adults, with MA exhibiting the highest prevalence (14.0%; 12.1–15.9) and NHW the lowest (6.5%; 5.9–7.2) (Fig. 4; Supplemental Table 4).

In the no-activity group, NHB had the highest CKD prevalence (22.3%; 20.3–24.2), while the lowest prevalence was observed in Other Race (14.3%; 11.7–16.9). Among those with some/irregular-activity, NHB remained highest (20.0%; 16.8–23.1), whereas OH had the lowest prevalence (10.9%; 7.5–14.3). For regularly active adults, NHB again showed the highest prevalence (17.3%; 15.9–18.7), and NHW had the lowest (10.0%;9.2–10.8) (Fig. 4; Supplemental Table 4).

Among no-activity group, NHB had the highest CVD prevalence (13.3%; 11.7–14.8), and MA had the lowest (7.4%; 6.0–8.8). For some/irregular-activity, NHB maintained the highest prevalence (10.1%; 8.2–12.0), while MA had the lowest (4.4%; 2.2–6.7). In the regular-activity group, NHB continued to lead (8.0%; 6.9–9.2), and OH had the lowest prevalence (6.0%; 4.6–7.3) (Fig. 4; Supplemental Table 4).

### 3.5. Complete-case selection assessment

To assess potential selection bias from complete-case analysis, excluded participants (n = 6442) were compared with included participants (n = 26,380; Supplemental Table 5). Excluded participants were slightly older (mean age 50.8 vs 49.5 years), more likely to be female (55%vs 51%), and had higher proportions of racial and ethnicity minority groups, including Non-HB (24%vs 21%), MA (17%vs 14%), and OH (13%vs 11%) participants. Excluded participants also had lower educational attainment (33%vs 23% with less than high school education), lower employment status (50%vs 56%), and lower income (28%vs 21% in the low-income category) (Supplemental Table 5).

### 3.6. Sensitivity analyses

Sensitivity analyses yielded largely consistent results with the primary analysis. When diabetes was redefined using an objective biomarker (glycohemoglobin ≥6.5%), associations between physical activity and diabetes remained directionally consistent across all models. However, one notable difference emerged in Model 4: the association for some/irregular-activity versus no-activity was non-significant in the primary analysis (aOR, 0.86; 95% CI, 0.71–1.04; *P* = 0.117) but reached statistical significance in the sensitivity analysis (aOR, 0.73; 95% CI, 0.61–0.88; *P* = 0.001) (Supplemental Table 6). When physical activity was operationalized as a binary goal-status measure, results were similarly consistent with the primary analysis. Participants in the At-goal group consistently demonstrated lower odds of CMDs compared to those in the Not-at-goal group across all three outcomes (all *P* < 0.05) (Supplemental Table 7). Finally, excluding BMI from the fully adjusted model produced results consistent with the primary analysis. Notably, however, the adjusted odds ratios in models without BMI were consistently lower than those in models retaining BMI across all three CMDs. When both BMI and depression score were additionally excluded, this pattern persisted across all outcomes, with further reduction in odds ratios. (Supplemental Table 8).

## 4. Discussion

This nationally representative study provides a comprehensive analysis of trends and disparities in CMD prevalence by physical activity level among U.S. adults from 2007 to 2018.

Diabetes prevalence increased significantly over the study period. This pattern aligns with the growing chronic disease burden in the U.S [25]. Meanwhile, the persistent significant inverse hierarchy across all survey cycles is consistent with PA being an important correlate of disease burden, even amid rising population-level trends [26]. CKD and CVD prevalence showed no clear linear trends, remaining relatively stable or fluctuating over the study period. This stability may be explained by improvements in risk factor control (e.g., hypertension and cholesterol management) and advances in treatment that offset rising obesity and diabetes prevalence [27,28]. Prior literature has documented plateauing CVD mortality in recent decades, suggesting that competing dynamics of prevention and treatment may underlie our findings [27,28]. A notable and concerning finding was that the significant increase in diabetes prevalence was observed not only in the overall population but also among adults reporting regular PA. This suggests that the drivers of the rising diabetes epidemic are multifactorial and extend beyond physical inactivity alone. Factors such as the increasing prevalence of obesity, changes in dietary patterns towards ultra-processed foods, and other environmental determinants may be exerting a population-level effect that is not fully mitigated by individual-level adherence to PA guidelines [29,30]. This underscores the critical need for broad public health strategies that address these upstream determinants in addition to promoting individual behavior change.

After full adjustment, regular-activity was associated with lower odds of all three CMDs, whereas some/irregular-activity was associated with lower odds of CVD only. This indicates that for diabetes and CKD, only guideline-adherent PA levels are associated with significantly lower odds after accounting for confounders like BMI and lifestyle factors [29,30]. In contrast, for CVD, even sub-guideline levels of activity ("Some/irregular-activity") were associated with lower odds relative to no-activity, highlighting a clinically relevant implication for populations struggling to meet full guidelines [31]. This finding may reflect biological mechanisms: PA improves endothelial function, reduces inflammation, and modulates autonomic tone even at sub-threshold levels, whereas glycemic control and renal protection may require higher-intensity or more sustained activity.

Racial and ethnic disparities persisted across all PA levels: non-Hispanic Black adults had the highest prevalence of all three CMDs in the no-activity group, while Mexican American adults showed the highest diabetes prevalence among those reporting some or regular activity. These findings underscore that PA's benefits are not uniformly experienced across racial and ethnic groups [32-34]. The complex patterns highlight the need for context-specific strategies [35]. Although structural inequities, such as neighborhood safety, access to recreational facilities, and healthcare accessibility, are plausible explanations based on the existing literature [35], these factors were not directly measured in this study and therefore remain speculative.

These findings carry several important public health implications. First, they reinforce PA as a cornerstone of chronic disease prevention. However, they also highlight that messaging should go beyond the binary of “meeting vs. not meeting guidelines.” While guideline-level PA is optimal for diabetes and CKD prevention, even modest activity is associated with benefits for cardiovascular health, providing an achievable entry point for sedentary individuals. Second, interventions must account for persistent racial and ethnic disparities. Standardized PA promotion alone may be insufficient if structural inequities—such as neighborhood safety, access to recreational facilities, cultural norms, and healthcare accessibility—are not addressed [35]. Community-based, culturally tailored interventions that combine PA promotion with efforts to address social determinants of health are essential. Third, surveillance systems should continue monitoring both overall CMD trends and subgroup-specific patterns, as rising diabetes prevalence and stagnant disparities underscore the ongoing challenges in achieving equity in chronic disease prevention [25,33,35].

Our study also adds to the broader evidence base on the dose–response relationship between PA and CMD outcomes. While some research suggests diminishing returns beyond guideline levels, our results indicate that benefits for diabetes and CKD may only be evident at or above recommended thresholds [29,30]. Future work should evaluate whether intensity, type, or accumulated patterns of activity differentially influence outcomes. For instance, resistance training may be particularly effective in glycemic control, while aerobic activity may confer greater cardiovascular benefit [31]. Longitudinal studies integrating objective PA measures could clarify whether certain types or patterns of sub-threshold activity might still offer protective effects against metabolic and renal diseases [29,30].

This study has several strengths. The use of six consecutive NHANES cycles (2007–2018) with a nationally representative sample of 26,380 US adults supports the generalizability of findings. The integration of three analytical dimensions-temporal trends, PA-CMD associations with sequential covariate adjustment, and racial/ethnic disparities—provides a comprehensive assessment of the evolving CMD landscape. The application of survey-weighted methods, age-standardization, formal interaction tests, and multiple sensitivity analyses strengthens the robustness of the results.

Despite its strengths, including the use of a nationally representative dataset and comprehensive adjustment for covariates, this study has several limitations. First, the use of cross-sectional data precludes drawing causal inference from PA-CMD associations and reverse causality is possible. Second, the interpretation of temporal trends is limited; observed prevalence changes could reflect cohort effects, diagnostic shifts, or changes in health-seeking behaviors rather than true incidence changes. However, we did account for age by applying age-standardization and adjusting for age in multivariable models, which helps reduce potential bias due to shifts in the underlying age distribution across survey cycles. Despite this, other temporal changes that we could not directly address such as evolving diagnostic criteria or access to car may still influence the observed trends. Third, PA was self-reported, which is prone to measurement error; thus, some misclassification of PA level is likely, and objective activity data might have yielded different results. Similarly, two of the CMD outcomes (diabetes and CVD) were defined by self-report of a diagnosis, which could underestimate true prevalence due to undiagnosed cases or recall issues. Moreover, the categorization of PA into three levels may oversimplify PA behavior, as information on intensity patterns, bout duration, and domain (occupational vs. leisure) was not captured. Fourth, although we adjusted for a wide range of covariates, residual confounding by unmeasured or imperfectly measured factors cannot be entirely ruled out. For instance, dietary patterns, genetic predispositions, or environmental factors not captured by NHANES could influence both PA levels and CMD risk. Fifth, Complete-case analysis resulted in the exclusion of 6442 participants due to missing covariate data. Comparisons between included and excluded participants revealed systematic differences in age, sex, race/ethnicity, educational level, employment status, and income, suggesting that findings may be less generalizable to socioeconomically disadvantaged populations. Sixth, stratified analyses by race/ethnicity were descriptive and should be interpreted with caution, as certain subgroups - particularly the 'Other race' category-had relatively small sample sizes, which may result in unstable prevalence estimates and wider confidence intervals. Seventh, as odds ratios may overestimate true prevalence ratios when outcome prevalence is common, readers are cautioned against directly interpreting the reported ORs as relative risks. Finally, while NHANES is representative of the U.S. civilian noninstitutionalized population, findings may not be generalizable to institutionalized populations or other countries. Future research should consider these limitations, perhaps incorporating objective PA data, employing longitudinal designs, and exploring more granular definitions of PA to further elucidate these complex relationships.

## 5. Conclusion

This NHANES study (2007–2018) reveals concerning increases in diabetes prevalence among U.S. adults over the study period. Crucially, we observed a consistent inverse hierarchy in CMD prevalence across all CMDs, where meeting PA guidelines was associated with the lowest prevalence. Notably, even some PA defined as activity insufficient to meet full guideline recommendations was associated with lower odds of CVD. These findings also uncovered significant and persistent racial and ethnic disparities in CMD burden across all activity levels. These results underscore the necessity of not only promoting guideline-adherent PA but also implementing tailored, interventions that address the specific barriers faced by populations experiencing a higher burden of CMD despite PA, in order to mitigate the rising disparity in CMD burden.

## Supplementary Material

1

2

Supplementary material associated with this article can be found, in the online version, at doi:10.1016/j.ahr.2026.100290.

## Figures and Tables

**Fig. 1.**
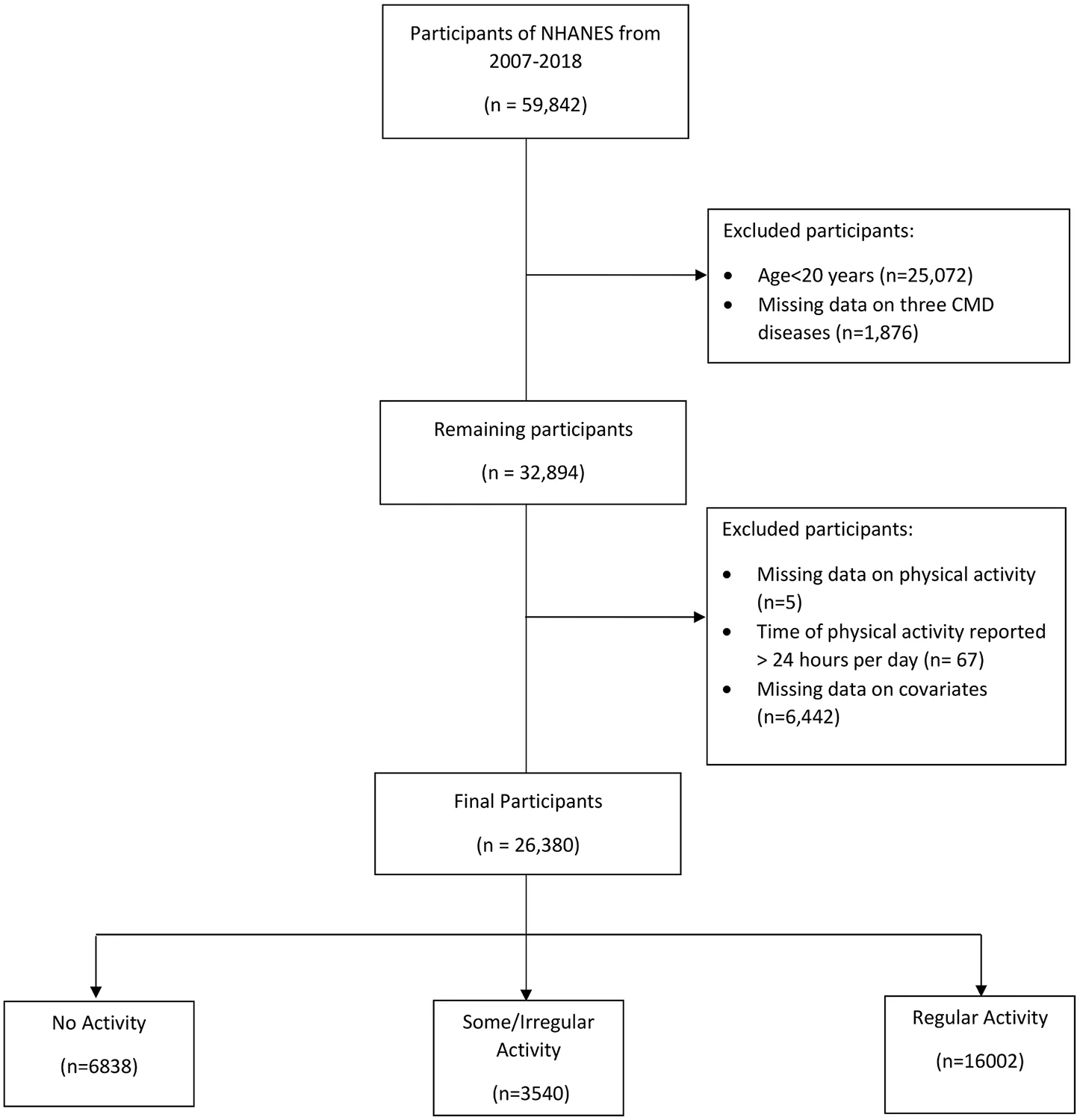
Sample creation flowchart (National Health and Nutrition Examination Survey, 2007–2018).

**Fig. 2.**
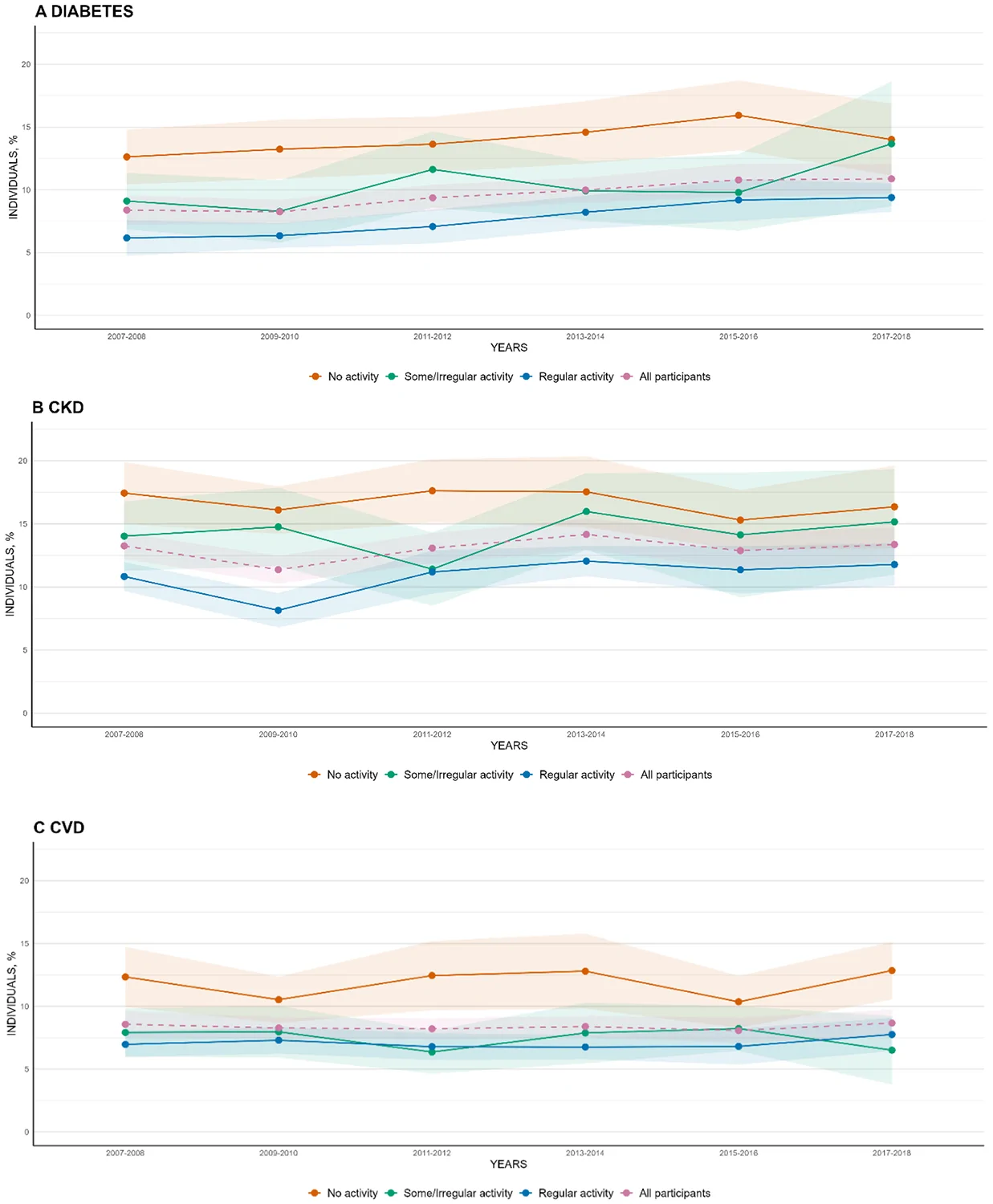
Age-Standardized Prevalence Trends of Cardiometabolic Diseases by Physical Activity Level Among U.S. Adults, NHANES 2007–2018. (A) Diabetes, (B) CKD, and (C) CVD. Each line represents a physical activity subgroup: orange = No activity; green = Some/Irregular activity; blue = Regular activity; pink dashed = All participants (overall trend). The x-axis represents six consecutive two-year NHANES survey cycles from 2007–2008 to 2017–2018, and the y-axis represents the percentage of individuals within each group or subgroup. The shaded bands represent 95% confidence intervals. CKD - Chronic kidney disease; CVD - Cardiovascular disease.

**Fig. 3.**
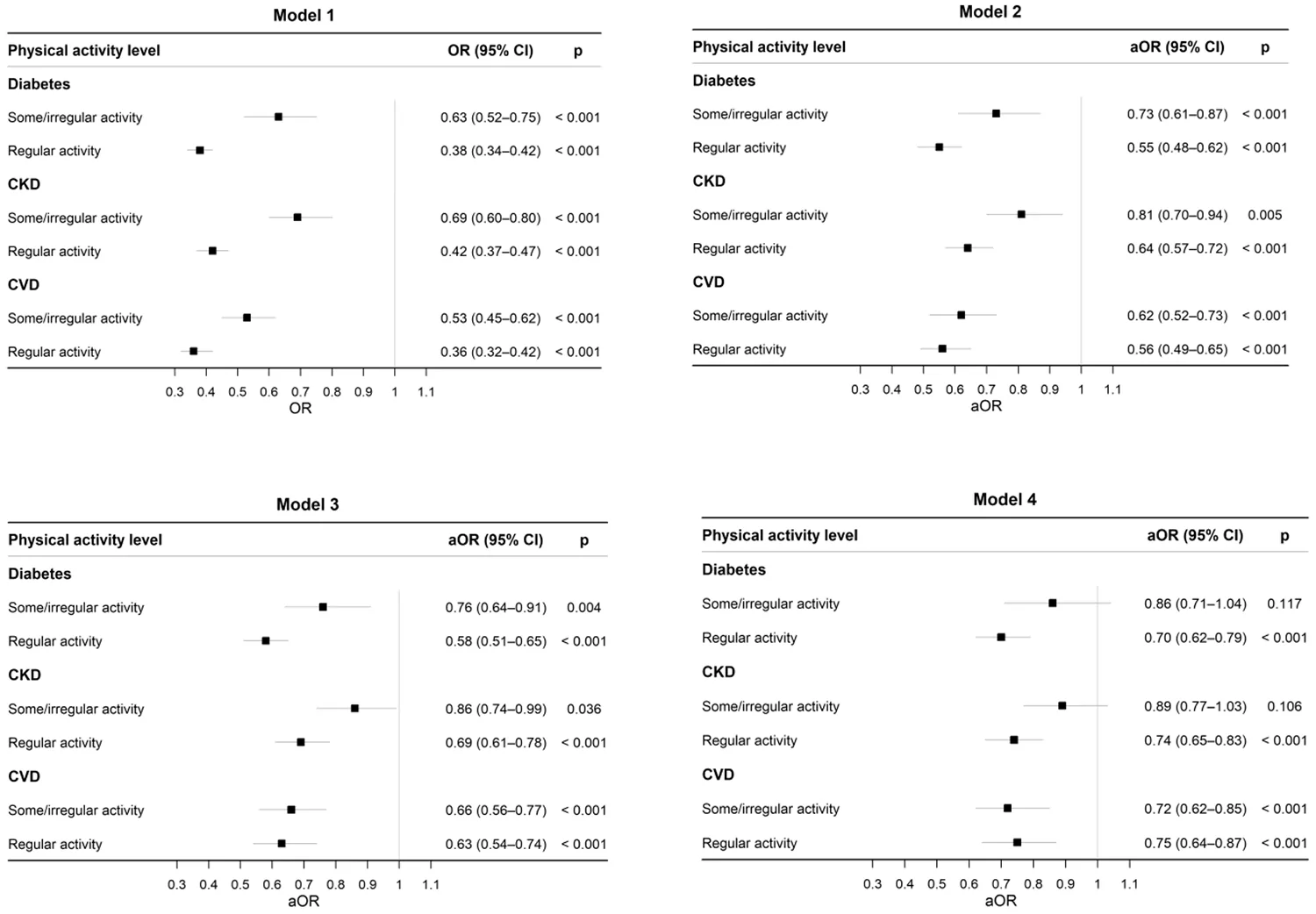
Survey-Weighted Associations Between Physical Activity and CMD Diseases. Survey-weighted multivariable logistic regression was utilized. Model 1 presents the bivariate association between physical activity groups and diabetes, CKD, and CVD, using the "No activity" group as the reference. Model 2 was adjusted for age, race/ethnicity (race and Hispanic origin), and sex. Model 3 was further adjusted for education, marital status, employment, and the ratio of income to poverty. Model 4 was further adjusted for BMI, depression score, alcohol use status, and smoking status. Design-based Nagelkerke pseudo-R^2^ values for each sequential model were as follows - Diabetes: Model 1 = 0.03, Model 2 = 0.15, Model 3 = 0.16, Model 4 = 0.23; CKD: Model 1 = 0.03, Model 2 = 0.17, Model 3 = 0.18, Model 4 = 0.19; CVD: Model 1 = 0.04, Model 2 = 0.24, Model 3 = 0.27, Model 4 = 0.30. Interaction tests revealed statistically significant effect modification by race/ethnicity on the PA-CMD association for all three outcomes in the fully adjusted Model 4 (all *P* for interaction <.001), based on a Wald F-test of the PA × race/ethnicity interaction term. OR - Odds ratio; aOR - Adjusted odds ratio; CI - Confidence interval; Ref - Reference; CMD - Cardiometabolic disease; CKD - Chronic kidney disease; CVD - Cardiovascular disease.

**Fig. 4.**
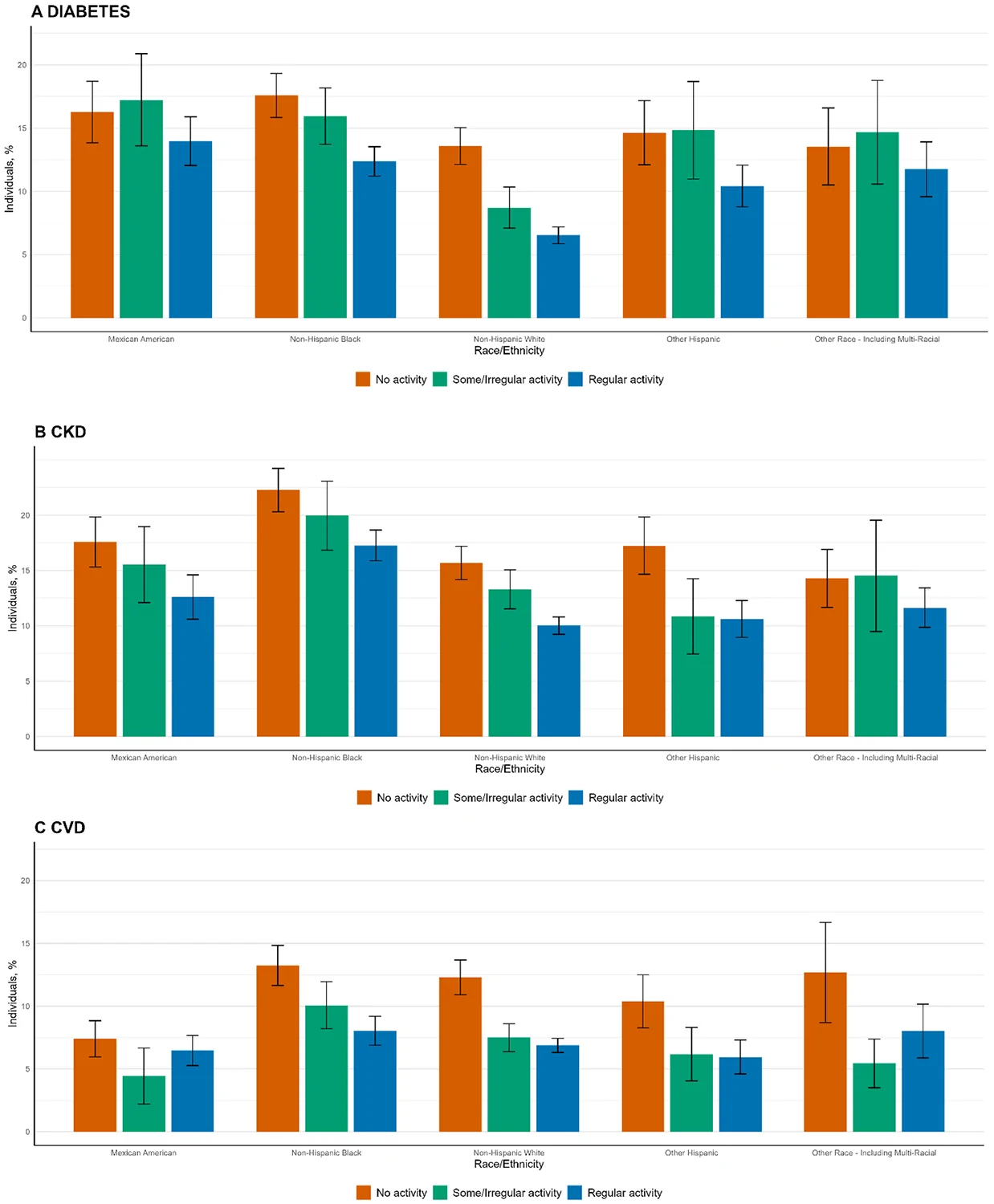
Age-Standardized Prevalence of Cardiometabolic Diseases by Physical Activity Level and Race/Ethnicity Among U.S. Adults, NHANES 2007–2018. (A) diabetes, (B) CKD, and (C) CVD. Bar colors represent physical activity level: orange = No activity, green = Some/Irregular activity, and blue = Regular activity. Error bars indicate 95% confidence intervals. The y-axis represents the percentage of individuals within each subgroup. NHANES 2007–2018. CKD - Chronic kidney disease; CVD - Cardiovascular disease.

**Table 1:** Characteristics of participants by physical activity (PA) groups (NHANES, 2007–2018).

Characteristic	No activity	Some/ Irregular activity	Regular activity	*p*
	n = 6838	n = 3540	n = 16,002	
**Age (years), Mean (SD)**	53.41 (17.13)	50.33 (16.37)	44.81 (16.24)	<0.001
**Sex, n (%)**				<0.001
Female	4129 (62)	2097 (61)	7192 (46)	
Male	2709 (38)	1443 (39)	8810 (54)	
**Race/Ethnicity (race and Hispanic origin), n (%)**				<0.001
Mexican American	1113 (9.50)	453 (6.70)	2227 (7.80)	
Non-Hispanic Black	1564 (13)	786 (11)	3189 (9.80)	
Non-Hispanic White	2744 (64)	1496 (69)	7162 (70)	
Other Hispanic	756 (6)	328 (4.8)	1539 (5.20)	
Other Race (including multiracial)	661 (7.50)	477 (8.50)	1885 (7.20)	
**Education level, n (%)**				<0.001
Less than high school	2172 (23)	754 (13)	3075 (12)	
High school graduate	1682 (26)	818 (23)	3568 (22)	
Some college or above	2984 (52)	1968 (64)	9359 (66)	
**Employment status, n (%)**				<0.001
Employed	2914 (51)	1820 (59)	10,161 (69)	
Unemployed	3924 (49)	1720 (41)	5841 (31)	
**Ratio of family income to poverty, n (%)**				<0.001
Low	1663 (18)	727 (13)	3184 (14)	
Middle	3867 (54)	1871 (47)	8314 (47)	
High	1308 (28)	942 (40)	4504 (39)	
**Marital status, n (%)**				0.017
Married	3514 (55)	1895 (58)	8130 (55)	
Otherwise	3324 (45)	1645 (42)	7872 (45)	
**BMI (kg/m^2^), n (%)**				<0.001
<18.5	104 (2)	57 (2)	227 (1)	
18.5–24.9	1483 (20)	903 (25)	4648 (30)	
25–29.9	2130 (30)	1126 (32)	5345 (34)	
≥30	3121 (48)	1454 (41)	5782 (35)	
**Depression score, Mean (SD)**	3.98 (4.78)	3.27 (4.28)	2.76 (3.78)	<0.001
**Drinking status, n (%)**				<0.001
Never drinkers	1213 (14)	518 (10)	1807 (8)	
Former drinkers	1739 (23)	699 (16)	2401 (13)	
Current drinkers	3886 (63)	2323 (74)	11,794 (79)	
**Smoking status, n (%)**				0.044
Never smokers	3669 (54)	1973 (56)	8887 (56)	
Former smokers	1787 (26)	907 (26)	3765 (24)	
Current smokers	1382 (20)	660 (18)	3350 (20)	
**Diabetes (non-age-standardized), n (%)**				
Yes	1383 (17)	545 (11)	1545 (7)	<0.0001
**CKD (non-age-standardized), n (%)**				
Yes	1770 (21)	662 (16)	2036 (10)	<0.001
**CVD (non-age-standardized), n (%)**				
Yes	1193 (15)	401 (9)	1246 (6)	<0.001

SD - standard deviation; BMI - Body mass index; CKD - Chronic kidney disease; CVD - Cardiovascular disease.

Note: Participants were classified as having no activity, some/irregular activity, or meeting (regular activity) the physical activity recommendations (≥150 min of moderate-intensity or ≥75 min of vigorous-intensity activity per week).

Categorical variables are presented as unweighted counts (n) and weighted percentages. Continuous variables are presented as weighted means and standard deviations. p-Values were derived from weighted analyses (Rao-Scott χ^2^ or design-adjusted Wald F-tests) to account for NHANES' complex survey design and ensure nationally representative inferences.

**Table 2:** Pattern of change in CMD prevalence across NHANES cycles, 2007–2018.

	Diabetes				CKD				CVD			
	Δ pp ^a^	OR	95% CI	*p*	Δ pp ^b^	OR	95% CI	*p*	Δ pp ^b^	OR	95% CI	*p*
**2007–2008**	—	*Ref*			—	*Ref*			—	*Ref*		
**2009–2010**	−0.14	1.00	(0.79, 1.27)	0.985	−1.89	0.87	(0.75, 1.02)	0.076	−0.29	1.01	(0.78, 1.30)	0.930
**2011–2012**	+0.98	1.18	(0.92, 1.51)	0.188	−0.18	1.03	(0.87, 1.21)	0.743	−0.36	1.01	(0.82, 1.24)	0.930
**2013–2014**	+1.60	1.27	(1.01, 1.60)	0.041	+0.90	1.16	(1.00, 1.34)	0.055	−0.18	1.07	(0.86, 1.33)	0.535
**2015–2016**	+2.40	1.41	(1.08, 1.84)	0.013	−0.38	1.06	(0.88, 1.27)	0.553	−0.49	1.05	(0.83, 1.32)	0.683
**2017–2018**	+2.49	1.45	(1.16, 1.80)	0.001	+0.11	1.11	(0.96, 1.30)	0.164	+0.10	1.15	(0.89, 1.48)	0.291
**Global trend test** ^b^	—	1.09	(1.05, 1.13)	< 0.001	—	1.04	(1.01, 1.06)	0.015	—	1.03	(0.98, 1.07)	0.252

Δpp - Change in percentage points; OR - Odds ratio; CI - Confidence interval; Ref - Reference; CMD - Cardiometabolic disease.

Note. Survey-weighted logistic regression was utilized to estimate prevalence differences across NHANES cycle, with the 2007–2008 cycle serving as the reference group.

a Absolute change in age-standardized weighted prevalence (percentage points) relative to the 2007–2008 reference cycle, derived using the delta method with 2010 U.S. Census age-standardization weights (age groups: 20–44, 45–64, ≥65 years).

b Global trend tests were assessed by modeling survey cycle as a continuous ordinal variable (coded 1 = 2007–2008 through 6 = 2017–2018). The OR represents the odds change per one-cycle increment (approximately two years).
