# Supplementary material for: Trends and disparities in cardiometabolic disease prevalence by physical activity among US adults: NHANES 2007–2018
Source: Aging Health Res. Author manuscript; Available in PMC 2026 Oct 14. (PMC13660780; doi:10.1016/j.ahr.2026.100290)
Supplement: Supplementary file 1 [file NIHMS2210205-supplement-2.pdf]

Authorship change request

Important information. Please read before completing this form.
This form is to request any change in authorship (additions, removals, or reordering) after the submission of a manuscript, including changes in corresponding authors, if any. This form should not be used for changes requested *after* publication or for name changes or corrections.

Prior to completing this form, all authors should carefully review the 'Duties of Authors' section of the Elsevier publishing ethics policy, and in particular, the sections on:

- Authorship of the paper
- The use of generative AI and AI-assisted technologies in scientific writing and in figures, images and artwork

Please also carefully review the journal's guide for authors (this might also be referred to as 'instructions for authors') as some journals may have additional authorship criteria (e.g., the ICMJE guidelines for authorship).

The publisher and editor cannot investigate or mediate any authorship disputes. If you are unable to obtain agreement from all authors, including those you intend to remove, we recommend seeking guidance from your institution. We will not consider your change request and will not proceed with the publication of your manuscript until all outstanding authorship disputes are resolved.

If your manuscript is still under consideration, this completed form should be submitted in Editorial Manager for consideration as part of your revision submission (use the 'cover letter' file type).

If your manuscript has already been accepted and is in the proofing or production stages, please return this completed form to the Journal Manager.

If the final version of your manuscript has already been published, a corrigendum will be required. Please see the Article Correction, Retraction and Removal Policy.

Section 1. Submission information

To be completed by the corresponding author.

**Submission information**	
**Journal title**	Aging and Health Research
**Manuscript number and/or article number**	AHR-D-26-00037R1
**Manuscript title**	Trends and Disparities in Cardiometabolic Disease Prevalence by Physical Activity Among US Adults:

**Change(s) requested** (indicate as appropriate)	
☐ Add new author(s)	☐ Remove author(s)
☑ Change the corresponding author	☐ Change the order of authors

Section 2. Author(s) added or removed

Complete one table for each author to be added or removed. Please include as much detail as possible in the "Reason for change" section so that we can evaluate if the change is necessary. At a minimum, this should include an explanation for why the change is being requested and why the author was/was not included in the original author list.

If the form is not provided, incomplete, or the reasons provided are insufficiently detailed or do not address the points above, your request will be denied and your submission may be rejected.

Unless instructed by the editor, further changes to the author list without an approved authorship change request will result in the rejection of your submission, or retraction, if the article has already been published.

2.1 Author information

**Given/first name(s)**	Zhijing
**Family/last name**	Xu
**Email address**	xuzh@montclair.edu
**Institution**	

**Change(s) requested** (indicate as appropriate)	☐ Add new author	☐ Remove author	☑ Make the corresponding author

Individual contributions per CRediT Contributor Roles Taxonomy (required for author additions only)

☑ Conceptualization	☑ Data curation	☑ Formal analysis
☑ Funding acquisition	☑ Investigation	☑ Methodology
☑ Project administration	☑ Resources	☑ Software
☑ Supervision	☑ Validation	☑ Visualization
☑ Writing – original draft	☑ Writing – review & editing	

Reason for the change

Dr. Xu has been acting as co-corresponding author handing the submission and editorial correspondence. Due to an editing oversight, Dr. Xu's co-corresponding author designation was inadvertently omitted in the manuscript text, which we now request to correct.

2.2 Author information

**Given/first name(s)**	
**Family/last name**	
**Email address**	
**Institution**	
**Change(s) requested** (indicate as appropriate)	☐ Add new author ☐ Remove author ☐ Make the corresponding author

Individual contributions per CRediT Contributor Roles Taxonomy (required for author additions only)

☐ Conceptualization	☐ Data curation	☐ Formal analysis
☐ Funding acquisition	☐ Investigation	☐ Methodology
☐ Project administration	☐ Resources	☐ Software
☐ Supervision	☐ Validation	☐ Visualization
☐ Writing – original draft	☐ Writing – review & editing	

Reason for the change

2.3 Author information

**Given/first name(s)**	
**Family/last name**	
**Email address**	
**Institution**	

**Change(s) requested** (indicate as appropriate)	☐	Add new author	☐	Remove author	☐	Make the corresponding author

Individual contributions [per CRediT Contributor Roles Taxonomy] (required for author additions only)

☐	Conceptualization	☐	Data curation	☐	Formal analysis
☐	Funding acquisition	☐	Investigation	☐	Methodology
☐	Project administration	☐	Resources	☐	Software
☐	Supervision	☐	Validation	☐	Visualization
☐	Writing – original draft	☐	Writing – review & editing		

Reason for the change

2.4 Author information

**Given/first name(s)**	
**Family/last name**	
**Email address**	
**Institution**	
**Change(s) requested** (indicate as appropriate)	☐ Add new author ☐ Remove author ☐ Make the corresponding author

Individual contributions per CRediT Contributor Roles Taxonomy (required for author additions only)

☐ Conceptualization	☐ Data curation	☐ Formal analysis
☐ Funding acquisition	☐ Investigation	☐ Methodology
☐ Project administration	☐ Resources	☐ Software
☐ Supervision	☐ Validation	☐ Visualization
☐ Writing – original draft	☐ Writing – review & editing	

Reason for the change

2.5 Author information

**Given/first name(s)**	
**Family/last name**	
**Email address**	
**Institution**	
**Change(s) requested** (indicate as appropriate)	☐ Add new author ☐ Remove author ☐ Make the corresponding author

Individual contributions per CRediT Contributor Roles Taxonomy (required for author additions only)

☐ Conceptualization	☐ Data curation	☐ Formal analysis
☐ Funding acquisition	☐ Investigation	☐ Methodology
☐ Project administration	☐ Resources	☐ Software
☐ Supervision	☐ Validation	☐ Visualization
☐ Writing – original draft	☐ Writing – review & editing	

Reason for the change

*Add additional page(s) as needed for more requested changes.

Section 3. Author order and agreement

Provide the author list in the order that you would like it to be published.

The form must be signed individually by each author, including any added/removed authors. In cases of consortia group authorship, the corresponding author may sign on behalf of the group.

While handwritten signatures are acceptable, we highly encourage the use of electronic signature software (DocuSign, Adobe Sign, HelloSign, or similar) with valid e-signatures. These signatures should reflect your institutional information and email, as provided in the author list below. **Typed signatures or images of signatures will not be accepted**.

By signing this form all authors agree:

1) that they have read and acknowledge the publishing ethics policies linked in the "Important Information" section of this form;
2) agree to the addition and/or removal of the authors listed in section 2 and to the revised order of the author list in this section 3, and;
3) that all information provided accurately reflects the authorship of the article.

Agreement of removed author(s)

Full name	Email address	Signature	Date

*Add additional page(s) as needed.

Proposed author list

Order	Full name	Email address	Signature	Date
01	Zhijing Xu	xuzh@montclair.edu	Zhi jing Xu	July 27, 2026
02	Jinyu Hu	jh7972@nyu.edu	Jinyu Hu	July 27, 2026
03	Jackie Finik	jackie.finik@nyu.edu	[signature]	July 27, 2026
04	Xiang Qi	xq450@nyu.edu	Xiang QI	July 27, 2026
05	Ruotong Liu	liu.r@nyu.edu	[signature]	July 27, 2026
06	Yaguang Zheng	yaguang.zheng@nyu.edu	[signature]	July 27, 2026
07	Bei Wu	bei.wu@nyu.edu	Bei Wu	July 27, 2026
08				
09				
10				
11				
12				
13				
14				
15				
16				
17				
18				
19				
20				
21				
22				
23				
24				
25				

*Add additional page(s) as needed.
