# Supplementary material for: Trends and disparities in cardiometabolic disease prevalence by physical activity among US adults: NHANES 2007–2018
Source: Aging Health Res. Author manuscript; Available in PMC 2026 Oct 14. (PMC13660780; doi:10.1016/j.ahr.2026.100290)
Supplement: Supplementary file 2 [file NIHMS2210205-supplement-1.docx]

**Supplemental Table 1**

*NHANES Variable Names, Labels, and Survey Cycles Corresponding to Study Variables Utilized in Analyses, 2007–2018*

| **Variable Name** | **Variable Label** | **2007-2008** | **2009-2010** | **2011-2012** | **2013-2014** | **2015-2016** | **2017-2018** |
| --- | --- | --- | --- | --- | --- | --- | --- |
| **Survey design variables** | | | | | | | |
|  |  | DEMO_E | DEMO_F | DEMO_G | DEMO_H | DEMO_I | DEMO_J |
| SEQN | Respondent sequence number | Y | Y | Y | Y | Y | Y |
| SDMVPSU | Masked Variance Pseudo-PSU | Y | Y | Y | Y | Y | Y |
| SDMVSTRA | Masked Variance Pseudo-Stratum | Y | Y | Y | Y | Y | Y |
| WTINT2YR | Full Sample 2 Year Interview Weight | Y | Y | Y | Y | Y | Y |
| WTMEC2YR | Full Sample 2 Year MEC Exam Weight | Y | Y | Y | Y | Y | Y |
| **Covariates** |  |  |  |  |  |  |  |
|  |  | DEMO_E | DEMO_F | DEMO_G | DEMO_H | DEMO_I | DEMO_J |
| RIDAGEYR | Age at Screening Adjudicated - Recode | Y | Y | Y | Y | Y | Y |
| RIAGENDR | Gender | Y | Y | Y | Y | Y | Y |
| RIDRETH1 | Race/Ethnicity - Recode | Y | Y | Y | Y | Y | Y |
| RIDRETH3 ^a^ | Race/Hispanic origin w/ NH Asian |  |  | Y | Y | Y | Y |
| DMDEDUC2 | Education Level - Adults 20+ | Y | Y | Y | Y | Y | Y |
| DMDMARTL | Marital Status | Y | Y | Y | Y | Y | Y |
| INDFMPIR | Ratio of family income to poverty | Y | Y | Y | Y | Y | Y |
|  |  | OCQ_E | OCQ_F | OCQ_G | OCQ_H | OCQ_I | OCQ_J |
| OCD150 | Type of work done last week | Y | Y | Y | Y | Y | Y |
|  |  | BMX_E | BMX_F | BMX_G | BMX_H | BMX_I | BMX_J |
| BMXBMI | Body Mass Index (kg/m^2^) | Y | Y | Y | Y | Y | Y |
|  |  | DPQ_E | DPQ_F | DPQ_G | DPQ_H | DPQ_I | DPQ_J |
| DPQ010 | Have Little interest in doing things | Y | Y | Y | Y | Y | Y |
| DPQ020 | Feeling down, depressed, or hopeless | Y | Y | Y | Y | Y | Y |
| DPQ030 | Trouble sleeping or sleeping too much | Y | Y | Y | Y | Y | Y |
| DPQ040 | Feeling tired or having little energy | Y | Y | Y | Y | Y | Y |
| DPQ050 | Poor appetite or overeating | Y | Y | Y | Y | Y | Y |
| DPQ060 | Feeling bad about yourself | Y | Y | Y | Y | Y | Y |
| DPQ070 | Trouble concentrating on things | Y | Y | Y | Y | Y | Y |
| DPQ080 | Moving or speaking slowly or too fast | Y | Y | Y | Y | Y | Y |
| DPQ090 | Thought you would be better off dead | Y | Y | Y | Y | Y | Y |
|  |  | ALQ_E | ALQ_F | ALQ_G | ALQ_H | ALQ_I | ALQ_J |
| ALQ101 | Had at least 12 alcohol drinks/1 yr? | Y | Y | Y | Y | Y |  |
| ALQ110 | Had at least 12 alcohol drinks/lifetime? | Y | Y | Y | Y | Y |  |
| ALQ120Q | How often drink alcohol over past 12 mos | Y | Y | Y | Y | Y |  |
| ALQ111 ^b^ | Ever had a drink of any kind of alcohol |  |  |  |  |  | Y |
| ALQ121 ^b^ | Past 12 mo how often have alcohol drink |  |  |  |  |  | Y |
| ALQ130 ^b^ | Avg number alcohol drinks/day - past 12 mos |  |  |  |  |  | Y |
|  |  | SMQ_E | SMQ_F | SMQ_G | SMQ_H | SMQ_I | SMQ_J |
| SMQ020 | Smoked at least 100 cigarettes in life | Y | Y | Y | Y | Y | Y |
| SMQ040 | Do you now smoke cigarettes | Y | Y | Y | Y | Y | Y |
| **Primary predictor – Physical activity** | | | | | | | |
|  |  | PAQ_E | PAQ_F | PAQ_G | PAQ_H | PAQ_I | PAQ_J |
| PAQ605 | Vigorous work activity | Y | Y | Y | Y | Y | Y |
| PAQ610 | Days vigorous work | Y | Y | Y | Y | Y | Y |
| PAD615 | Minutes vigorous-intensity work | Y | Y | Y | Y | Y | Y |
| PAQ620 | Moderate work activity | Y | Y | Y | Y | Y | Y |
| PAQ625 | Number of days moderate work | Y | Y | Y | Y | Y | Y |
| PAD630 | Minutes moderate-intensity work | Y | Y | Y | Y | Y | Y |
| PAQ635 | Walk or bicycle | Y | Y | Y | Y | Y | Y |
| PAQ640 | Number of days walk or bicycle | Y | Y | Y | Y | Y | Y |
| PAD645 | Minutes walk/bicycle for transportation | Y | Y | Y | Y | Y | Y |
| PAQ650 | Vigorous recreational activities | Y | Y | Y | Y | Y | Y |
| PAQ655 | Days vigorous recreational activities | Y | Y | Y | Y | Y | Y |
| PAD660 | Minutes vigorous recreational activities | Y | Y | Y | Y | Y | Y |
| PAQ665 | Moderate recreational activities | Y | Y | Y | Y | Y | Y |
| PAQ670 | Days moderate recreational activities | Y | Y | Y | Y | Y | Y |
| PAD675 | Minutes moderate recreational activities | Y | Y | Y | Y | Y | Y |
| **Outcome variables – Diabetes** | | | | | | | |
|  |  | DIQ_E | DIQ_F | DIQ_G | DIQ_H | DIQ_I | DIQ_J |
| DIQ010 | Doctor told you have diabetes | Y | Y | Y | Y | Y | Y |
|  |  | GHB_E | GHB_F | GHB_G | GHB_H | GHB_I | GHB_J |
| LBXGH | Glycohemoglobin (%) | Y | Y | Y | Y | Y | Y |
| **Outcome variables – CKD** | | | | | | | |
|  |  | BIOPRO_E | BIOPRO_F | BIOPRO_G | BIOPRO_H | BIOPRO_I | BIOPRO_J |
| LBXSCR | Creatinine (mg/dL) | Y | Y | Y | Y | Y | Y |
|  |  | ALB_CR_E | ALB_CR_F | ALB_CR_G | ALB_CR_H | ALB_CR_I | ALB_CR_J |
| URXUMA | Albumin, urine (ug/mL) | Y | Y | Y | Y | Y | Y |
| URXUCR | Creatinine, urine (mg/dL) | Y | Y | Y | Y | Y | Y |
| URDACT | First albumin creatinine ratio (mg/g) |  | Y | Y | Y | Y | Y |
| **Outcome variables – CVD** | | | | | | | |
|  |  | MCQ_E | MCQ_F | MCQ_G | MCQ_H | MCQ_I | MCQ_J |
| MCQ160B | Ever told had congestive heart failure | Y | Y | Y | Y | Y | Y |
| MCQ160C | Ever told you had coronary heart disease | Y | Y | Y | Y | Y | Y |
| MCQ160D | Ever told you had angina/angina pectoris | Y | Y | Y | Y | Y | Y |
| MCQ160E | Ever told you had heart attack | Y | Y | Y | Y | Y | Y |
| MCQ160F | Ever told you had a stroke | Y | Y | Y | Y | Y | Y |

CKD - Chronic kidney disease; CVD - Cardiovascular disease; BMI - Body mass index; PSU - Primary sampling unit; MEC - Mobile examination center; NH - Non-Hispanic; yr - Year; mos - Months; min - Minute; kg - Kilogram; m - Meter; mg - Milligram; dL - Deciliter; mL - Milliliter; µg - Microgram; g - Gram.

Note. Cycle-specific dataset suffixes follow NHANES naming conventions: E, 2007-2008; F, 2009-2010; G, 2011-2012; H, 2013-2014; I, 2015-2016; J, 2017–2018.

"Y" indicates that the variable was available and used in the corresponding survey cycle

^a^ RIDRETH3 was introduced beginning in the 2011-2012 cycle to distinguish non-Hispanic Asian participants and was therefore not available in earlier cycles.

^b^ Beginning in the 2017-2018 cycle, the alcohol consumption variables used in this study differ from those in prior cycles; ALQ101, ALQ110, and ALQ120Q were no longer available and ALQ111, ALQ121, and ALQ130 were used in their place, respectively.

**Supplemental Table 2**

***Population Distribution by Selected Age Groups in the 2010 U.S. Census (Used for Age Standardization in Prevalence Analysis)***

| **Age groups** | **Number** | **%** |
| --- | --- | --- |
| 20 to 44 years | 103,720,553 | 46.0 |
| 45 to 64 years | 81,489,445 | 36.1 |
| 65 years and over | 40,267,984 | 17.9 |
| Total | 225,477,982 | 100.0 |

Note. Population counts were derived from the U.S. Census Bureau 2010 Census Summary File 1 and used as standard weights for age standardization of CMD prevalence estimates.

**Supplemental Table 3**

*Age-Standardized Weighted Prevalence (%) of Diabetes, CKD, and CVD by Physical Activity Level and Survey Cycle Among U.S. Adults Aged ≥20 Years, NHANES 2007–2018*

| **Disease** | **PA level** | **2007-2008** | **2009-2010** | **2011-2012** | **2013-2014** | **2015-2016** | **2017-2018** | **Diff in prevalence ^a^** |
| --- | --- | --- | --- | --- | --- | --- | --- | --- |
|  |  | **% (95% CI)** | **% (95% CI)** | **% (95% CI)** | **% (95% CI)** | **% (95% CI)** | **% (95% CI)** | **% (95% CI)** |
| **Diabetes** |  | | | | | | | |
|  | No activity | 12.63 (10.45,14.80) | 13.24 (10.88, 15.60) | 13.64 (11.43, 15.84) | 14.59 (12.09, 17.08) | 15.94 (13.15, 18.72) | 14.01 (11.16, 16.86) | 1.38 (-2.20, 4.97) |
|  | Some/Irregular activity | 9.11 (6.86, 11.35) | 8.29 (5.81, 10.77) | 11.63 (8.59, 14.66) | 9.92 (7.54, 12.30) | 9.79 (6.74, 12.83) | 13.67 (8.71, 18.63) | 4.56 (-0.88, 10.00) |
|  | Regular activity | 6.17 (4.76, 7.58) | 6.35 (5.38, 7.33) | 7.08 (5.73, 8.43) | 8.22 (6.91, 9.54) | 9.19 (7.53, 10.86) | 9.39 (8.25, 10.52) | 3.22 (1.41, 5.02) |
|  | All participants | 8.39 (7.24, 9.55) | 8.25 (7.29, 9.21) | 9.37 (8.34, 10.41) | 9.99 (9.01, 10.98) | 10.79 (9.47, 12.11) | 10.88 (9.72, 12.05) | 2.49 (0.85, 4.13) |
| **CKD** |  | | | | | | | |
|  | No activity | 17.44 (14.99, 19.89) | 16.10 (14.21, 17.98) | 17.62 (15.11, 20.13) | 17.54 (14.74, 20.35) | 15.30 (12.92, 17.68) | 16.35 (13.08, 19.62) | -1.09 (-5.18, 2.99) |
|  | Some/Irregular activity | 14.03 (11.29, 16.78) | 14.76 (11.65, 17.88) | 11.41 (8.51, 14.31) | 15.98 (12.94, 19.03) | 14.13 (9.18, 19.07) | 15.16 (10.98, 19.33) | 1.13 (-3.87, 6.12) |
|  | Regular activity | 10.83 (9.70, 11.96) | 8.15 (6.79, 9.51) | 11.19 (9.48, 12.90) | 12.05 (10.86, 13.25) | 11.36 (9.49, 13.22) | 11.78 (10.11, 13.46) | 0.95 (-1.07, 2.97) |
|  | All participants | 13.26 (12.23, 14.28) | 11.37 (10.28, 12.46) | 13.08 (11.89, 14.27) | 14.16 (12.93, 15.39) | 12.88 (11.43, 14.33) | 13.37 (12.03, 14.71) | 0.11 (-1.57, 1.80) |
| **CVD** |  | | | | | | | |
|  | No activity | 12.34 (9.94, 14.74) | 10.53 (8.69, 12.36) | 12.45 (9.70, 15.20) | 12.80 (9.82, 15.78) | 10.36 (8.29, 12.42) | 12.85 (10.55, 15.14) | 0.51 (-2.81, 3.83) |
|  | Some/Irregular activity | 7.92 (6.00, 9.84) | 7.97 (5.91, 10.03) | 6.36 (4.66, 8.07) | 7.88 (5.47, 10.28) | 8.23 (6.46, 10.00) | 6.51 (3.79, 9.22) | -1.41 (-4,74, 1.91) |
|  | Regular activity | 6.97 (5.97, 7.97) | 7.30 (6.24, 8.36) | 6.79 (5.73, 7.85) | 6.76 (5.79, 7.72) | 6.81 (5.37, 8.24) | 7.76 (6.44, 9.07) | 0.79 (-0.87, 2.44) |
|  | All participants | 8.57 (7.53, 9.62) | 8.28 (7.45, 9.12) | 8.21 (7.39, 9.02) | 8.39 (7.55, 9.23) | 8.08 (7.08, 9.08) | 8.67 (7.60, 9.74) | 0.10 (-1.40, 1.59) |

PA - Physical activity; CI - Confidence interval; Diff – Difference; CKD - Chronic kidney disease; CVD - Cardiovascular disease;

Note. All estimates were age-adjusted to the 2010 U.S. Census standard population (age groups: 20–44, 45–64, ≥65 years) using direct standardization with survey weights.

^a^ Difference in prevalence represents the age-standardized weighted prevalence difference (percentage points) between the NHANES cycle 2017-2018 and the reference cycle (2007-2008), with 95% confidence intervals reported.

**Supplemental Table 4**

*Age-Standardized Weighted Prevalence (%) of Diabetes, CKD, and CVD by Physical Activity Level and Race/Ethnicity Among U.S. Adults Aged ≥20 Years, NHANES 2007-2018*

|  | **PA level** | **Mexican American** | **Non-Hispanic Black** | **Non-Hispanic White** | **Other Hispanic** | **Other Race (Including Multiracial)** |
| --- | --- | --- | --- | --- | --- | --- |
|  |  | **% (95% CI)** | **% (95% CI)** | **% (95% CI)** | **% (95% CI)** | **% (95% CI)** |
| **Diabetes** |  | | | | | |
|  | **No activity** | 16.28 (13.85, 18.71) | 17.59 (15.85, 19.33) | 13.59 (12.13, 15.05) | 14.64 (12.11, 17.18) | 13.55 (10.51, 16.60) |
|  | **Some/Irregular activity** | 17.24 (13.60, 20.89) | 15.95 (13.73, 18.18) | 8.72 (7.10, 10.35) | 14.84 (10.98, 18.69) | 14.68 (10.58, 18.78) |
|  | **Regular activity** | 13.98 (12.05, 15.90) | 12.37 (11.21, 13.54) | 6.53 (5.87, 7.19) | 10.43 (8.78, 12.08) | 11.75 (9.58, 13.92) |
| **CKD** |  | | | | | |
|  | **No activity** | 17.58 (15.32, 19.84) | 22.27 (20.31, 24.23) | 15.69 (14.19, 17.20) | 17.25 (14.66, 19.84) | 14.28 (11.66, 16.91) |
|  | **Some/Irregular activity** | 15.54 (12.10, 18.97) | 19.96 (16.84, 23.08) | 13.30 (11.54, 15.06) | 10.86 (7.46, 14.26) | 14.52 (9.49, 19.55) |
|  | **Regular activity** | 12.61 (10.60, 14.61) | 17.27 (15.89, 18.66) | 10.03 (9.24, 10.81) | 10.62 (8.96, 12.29) | 11.64 (9.86, 13.43) |
| **CVD** |  | | | | | |
|  | **No activity** | 7.40 (5.96, 8.84) | 13.25 (11.65, 14.84) | 12.29 (10.91, 13.68) | 10.38 (8.27, 12.50) | 12.68 (8.69, 16.67) |
|  | **Some/Irregular activity** | 4.44 (2.20, 6.67) | 10.07 (8.20, 11.95) | 7.49 (6.38, 8.60) | 6.17 (4.05, 8.30) | 5.45 (3.50, 7.39) |
|  | **Regular activity** | 6.47 (5.27, 7.67) | 8.04 (6.89, 9.19) | 6.88 (6.31, 7.44) | 5.95 (4.60, 7.31) | 8.02 (5.88, 10.16) |

PA - Physical activity; CI - Confidence interval; CKD - Chronic kidney disease; CVD - Cardiovascular disease.

Note. All estimates were age-adjusted to the 2010 U.S. Census standard population (age groups: 20–44, 45–64, ≥65 years) using direct standardization with survey weights. Cell estimates with unweighted sample sizes below 500 were noted due to potential instability - Other Hispanic/some irregular activity (n = 328) and Other Race (including multiracial)/some irregular activity (n = 477).

**Supplemental Table 5**

*Comparison of Included and Excluded Participants, NHANES 2007-2018*

|  | **Included** | **Excluded** | ***p ^a^*** |
| --- | --- | --- | --- |
|  | N = 26,380 | N = 6,442 |  |
|  | N (%) | N (%) |  |
| Age (years), Mean (SD) | 49.52 (17.59) | 50.78 (18.22) | <0.001 |
| Sex |  |  | <0.001 |
| Female | 13,418 (51) | 3,533 (55) |  |
| Male | 12,962 (49) | 2,909 (45) |  |
| Race/ethnicity |  |  | <0.001 |
| Mexican American | 3,793 (14) | 1,112 (17) |  |
| Non-Hispanic Black | 5,539 (21) | 1,533 (24) |  |
| Non-Hispanic White | 11,402 (43) | 1,915 (30) |  |
| Other Hispanic | 2,623 (11) | 824 (13) |  |
| Other Race (including multiracial) | 3,023 (11) | 1,058 (16) |  |
| Education level |  |  | <0.001 |
| Less than high school | 6,001 (23) | 2,127 (33) |  |
| High school graduate/GED or equivalent | 6,068 (23) | 1,405 (22) |  |
| Above high school | 14,311 (54) | 2,872 (45) |  |
| Employment status |  |  | <0.001 |
| Employed | 14,895 (56) | 3,211 (50) |  |
| Unemployed | 11,485 (44) | 3,212 (50) |  |
| Ratio of family income to poverty |  |  | <0.001 |
| Low | 5,574 (21) | 931 (28) |  |
| Middle | 14,052 (53) | 1,691 (51) |  |
| High | 6,754 (26) | 662 (20) |  |
| Marital status |  |  | 0.061 |
| Married | 13,539 (51) | 3,212 (50) |  |
| Otherwise | 12,841 (49) | 3,211 (50) |  |

SD - Standard deviation.

Note. Categorical variables are presented as unweighted counts (N) and unweighted percentages. Continuous variables are presented as unweighted means and standard deviations. Subgroup counts may not sum to the total sample size due to missing data for some variables.

^a^ *p*-Values were derived from independent samples t-tests for continuous variables and Pearson's chi-square tests for categorical variables.

**Supplemental Table 6**

*Sensitivity Analysis for Survey-Weighted Associations Between Physical Activity and Diabetes Using Glycohemoglobin as an Objective Outcome Measure, NHANES 2007-2018*

|  | **Model 1** | | |  | **Model 2** | | |  | **Model 3** | | |  | **Model 4** | | |
| --- | --- | --- | --- | --- | --- | --- | --- | --- | --- | --- | --- | --- | --- | --- | --- |
|  | **OR** | **95% CI** | ***p-Values*** |  | **aOR** | **95% CI** | ***p-Values*** |  | **aOR** | **95% CI** | ***p-Values*** |  | **aOR** | **95% CI** | ***p-Values*** |
| **Diabetes** | | | | | | | | | | | | | | | |
| No activity | *Ref* | | | | | | | | | | | | | | |
| Some/irregular activity | 0.54 | (0.45, 0.65) | < 0.001 |  | 0.63 | (0.52, 0.75) | < 0.001 |  | 0.65 | (0.54, 0.77) | < 0.001 |  | 0.73 | (0.61, 0.88) | 0.001 |
| Regular activity | 0.39 | (0.34, 0.44) | < 0.001 |  | 0.55 | (0.48, 0.62) | < 0.001 |  | 0.57 | (0.50, 0.65) | < 0.001 |  | 0.69 | (0.60, 0.79) | < 0.001 |
| Design-based Nagelkerke pseudo-R² | 0.03 | | |  | 0.14 | | |  | 0.14 | | |  | 0.22 | | |

OR - Odds ratio; aOR - Adjusted odds ratio; CI - Confidence interval; Ref - Reference.

Note. Diabetes was defined objectively as glycohemoglobin ≥ 6.5%. The analytical sample for the sensitivity analysis was derived from the primary analytical sample (n = 26,380); participants with missing glycohemoglobin values were further excluded, resulting in a sample of 25,543 participants; Survey-weighted multivariable logistic regression was used with "No activity" as the reference group. Model 1: unadjusted; Model 2: adjusted for age, sex, and race/ethnicity; Model 3: Model 2 plus education, marital status, employment, and income-to-poverty ratio; Model 4: Model 3 plus BMI, depression score, alcohol use, and smoking status.

**Supplemental Table 7**

*Sensitivity Analyses of Survey-Weighted Associations Between Physical Activity Goal Status and CMD Diseases*

|  | **Model 1** | | |  | **Model 2** | | |  | **Model 3** | | |  | **Model 4** | | |
| --- | --- | --- | --- | --- | --- | --- | --- | --- | --- | --- | --- | --- | --- | --- | --- |
|  | **OR** | **95% CI** | ***p-Values*** |  | **aOR** | **95% CI** | ***p-Values*** |  | **aOR** | **95% CI** | ***p-Values*** |  | **aOR** | **95% CI** | ***p-Values*** |
| **Diabetes** | | | | | | | | | | | | | | | |
| Not-at-goal | *Ref* | | | | | | | | | | | | | | |
| At-goal | 0.44 | (0.40, 0.49) | < 0.001 |  | 0.61 | (0.54, 0.68) | < 0.001 |  | 0.64 | (0.57, 0.71) | < 0.001 |  | 0.74 | (0.66, 0.82) | < 0.001 |
| Design-based Nagelkerke pseudo-R² | 0.03 | | |  | 0.15 | | |  | 0.16 | | |  | 0.23 | | |
| **CKD** | | | | | | | | | | | | | | | |
| Not-at-goal | *Ref* | | | | | | | | | | | | | | |
| At-goal | 0.47 | (0.43, 0.52) | < 0.001 |  | 0.69 | (0.62, 0.77) | < 0.001 |  | 0.73 | (0.66, 0.82) | < 0.001 |  | 0.77 | (0.68, 0.86) | < 0.001 |
| Design-based Nagelkerke pseudo-R² | 0.03 | | |  | 0.17 | | |  | 0.18 | | |  | 0.19 | | |
| **CVD** | | | | | | | | | | | | | | | |
| Not-at-goal | *Ref* | | | | | | | | | | | | | | |
| At-goal | 0.45 | (0.40, 0.50) | < 0.001 |  | 0.66 | (0.58, 0.75) | < 0.001 |  | 0.72 | (0.63, 0.83) | < 0.001 |  | 0.83 | (0.73, 0.94) | 0.004 |
| Design-based Nagelkerke pseudo-R² | 0.03 | | |  | 0.23 | | |  | 0.26 | | |  | 0.30 | | |

OR - Odds ratio; aOR - Adjusted odds ratio; CI - Confidence interval; Ref - Reference; CMD - Cardiometabolic disease; CKD - Chronic kidney disease; CVD - Cardiovascular disease.

Note. Participants were categorized into two groups: Not-at-goal, defined as not meeting physical activity guideline recommendations (< 150 min/week of moderate activity or < 75 min/week of vigorous activity); and At-goal, defined as meeting guideline recommendations (≥ 150 min/week of moderate activity or ≥ 75 min/week of vigorous activity). Survey-weighted multivariable logistic regression was utilized. Model 1 presents the bivariate association between goal status groups and diabetes, CKD, and CVD, using the "Not-at-goal" group as the reference. Model 2 was adjusted for age, race/ethnicity, and sex. Model 3 was further adjusted for education, marital status, employment, and the ratio of income to poverty. Model 4 was further adjusted for BMI, depression score, alcohol use status, and smoking status.

**Supplemental Table 8**

*Sensitivity Analyses of Survey-Weighted Associations Between Physical Activity and CMDs Excluding BMI and Depression Score as Covariates (Potential Mediators), NHANES 2007–2018*

|  | **Model 1** | | |  | **Model 2** | | |
| --- | --- | --- | --- | --- | --- | --- | --- |
|  | **aOR** | **95% CI** | ***p*** |  | **aOR** | **95% CI** | ***p*** |
| **Diabetes** |  | | |  |  |  |  |
| No activity | Ref  Ref | | | | | | |
| Some/irregular activity | 0.81 | (0.67, 0.97) | 0.020 |  | 0.78 | (0.65, 0.94) | 0.008 |
| Regular activity | 0.62 | (0.55, 0.71) | < 0.001 |  | 0.59 | (0.53, 0.67) | < 0.001 |
| Design-based Nagelkerke pseudo-R² | 0.17 | | |  | 0.17 | | |
| **CKD** |  | | |  |  | | |
| No activity | Ref | | | | | | |
| Some/irregular activity | 0.88 | (0.76, 1.01) | 0.069 |  | 0.87 | (0.75, 1.00) | 0.053 |
| Regular activity | 0.71 | (0.63, 0.81) | < 0.001 |  | 0.70 | (0.62, 0.80) | < 0.001 |
| Design-based Nagelkerke pseudo-R² | 0.18 | | |  | 0.18 | | |
| **CVD** |  | | |  |  | | |
| No activity | Ref | | | | | | |
| Some/irregular activity | 0.70 | (0.59, 0.82) | < 0.001 |  | 0.67 | (0.57, 0.79) | < 0.001 |
| Regular activity | 0.70 | (0.61, 0.82) | < 0.001 |  | 0.66 | (0.56, 0.76) | < 0.001 |
| Design-based Nagelkerke pseudo-R² | 0.29 | | |  | 0.28 | | |

OR - Odds ratio; aOR - Adjusted odds ratio; CI - Confidence interval; Ref - Reference; CMD - Cardiometabolic disease; CKD - Chronic kidney disease; CVD - Cardiovascular disease.

Note. Survey-weighted multivariable logistic regression was utilized with the "No activity" group as the reference. Model 1 was adjusted for age, sex, race/ethnicity , education, marital status, employment, ratio of income to poverty, alcohol use status, and smoking status, with BMI excluded given its potential role as a mediator in the pathway between physical activity and CMDs. Model 2 additionally excluded depression score, which may also function as a mediator in this pathway. Both models retained all remaining covariates from the fully adjusted primary model (Model 4).
